# Teacher-Identified Priorities and Resource Use in Government-Supported School Food and Nutrition Education in Korea

**DOI:** 10.3390/nu18172843

**Published:** 2026-08-30

**Authors:** Seung Jae Lee, Jieun Oh, Kyung Won Lee

**Affiliations:** 1 Department of Food and Nutrition, Yongin University, Yongin 17092, Republic of Korea; sjlee@yongin.ac.kr; 2 College of Science & Industry Convergence, Ewha Womans University, Seoul 03760, Republic of Korea; oje96@ewha.ac.kr; 3 Department of Home Economics Education, Korea National University of Education, Cheongju 28173, Republic of Korea

**Keywords:** food and nutrition education, educational needs assessment, Borich needs assessment, Locus for Focus model, adolescent dietary behaviors, government-supported programs

## Abstract

**Background/Objectives:** Government-supported school food and nutrition education depends on whether teachers can locate and use relevant programs and resources. This study identified teachers’ perceived priority topics and examined their awareness, use, and barriers regarding government resources in Korea. **Methods:** A nationwide voluntary online survey was completed by 1273 elementary, middle, and high school teachers between October and November 2024. Resource outcomes were analyzed in the full sample, whereas the educational needs assessment was limited to 575 teachers who had directly implemented a program supported by the Korean Ministry of Food and Drug Safety (MFDS). Participants rated the perceived importance and self-reported current implementation of 32 topics. Topic priorities were assessed using paired comparisons with false discovery rate adjustment, Borich scores, and the Locus for Focus model; exploratory subgroup and multivariable analyses examined variation in the importance–implementation gaps. **Results:** Perceived importance exceeded self-reported current implementation for 31 of the 32 topics after false discovery rate adjustment. The leading priorities included late night eating, adolescents and caffeine, healthier convenience store food choices, excessive weight control practices, and eating disorders. The overall importance–implementation gap was larger among elementary than middle/high school teachers (0.284 vs. 0.149, *p* < 0.001), and this difference remained after multivariable adjustment (B = −0.122, *p* = 0.001). Awareness and use were lower for the MFDS YouTube channel than for the food safety information website; lack of awareness was the most common reason for not using education-specific resources. **Conclusions:** Government agencies should combine classroom-ready, developmentally tailored content with active dissemination and teacher training. The identified priorities reflect the perception of teachers with direct implementation experience and should be complemented by future studies using observed implementation measures and student outcomes.

## 1. Introduction

School-based food and nutrition education is a critical strategy for establishing healthy dietary habits during childhood and adolescence, when lifelong food preferences and eating behaviors are formed [1]. International guidance conceptualizes school food and nutrition education as part of a whole school approach that connects curricula, food environments, school meals, and community engagement [2]. A recent scoping review identified recurrent barriers and enablers at teacher, classroom, school, and policy levels, including limited time, teacher preparation, curricular positioning, leadership, and access to usable resources [3]. Comparative studies have also documented variability in teachers’ nutrition knowledge, perceived roles, readiness, and use of nutrition education resources in Europe, Australia, the United States, China, South Africa, and Cyprus [4,5,6,7,8,9,10,11].

In Korea, food- and nutrition-related content is formally embedded primarily in elementary Practical Arts and in the Home Economics component of secondary Technology and Home Economics, while additional instruction may be organized through school-specific arrangements involving nutrition teachers and other staff [12,13]. This distributed delivery context makes it important to examine topic-specific needs and differences across school levels rather than assuming a single instructional model.

Needs assessment can distinguish topics that teachers consider important from those that they report implementing less fully. The Borich method provides an importance-weighted ranking of implementation gaps, whereas the Locus for Focus (LF) model displays each topic relative to the sample means for importance and gap [14,15]. Using both methods permits assessment of whether the same topics emerge under complementary criteria. Because both outputs are relative to the topics, scale, and sample studied, they identify priorities for planning rather than absolute thresholds of educational need or implementation difficulty.

Previous Korean studies have examined nutrition education tasks among nutrition teachers and school dietitians [13,16,17], and a recent study combined importance-performance comparisons, Borich scores, and the LF model to assess needs related to sustainable dietary education [18]. International work has usually examined teacher knowledge, readiness, barriers, or use of a particular resource [4,5,6,7,8,9,10,11]. Fewer studies have combined resource awareness and use with topic-level importance–implementation prioritization across elementary and secondary settings. This gap limits understanding of whether publicly provided resources align with the topics teachers find difficult to implement.

These questions are particularly relevant because the food environment encountered by Korean adolescents includes frequent late night eating, meal substitution with convenience store foods, high caffeine beverage consumption, and unhealthy weight control practices [19,20,21,22,23]. Such behaviors provide population level context for the educational topics, but they do not establish the needs of students in the participating schools. Addressing them may require practical decision-making, media and food label literacy, body image sensitive guidance, and skills for navigating readily available foods.

Against this background, the present study examined government-supported school food and nutrition education broadly, while using programs and resources supported by one national agency, the Korean Ministry of Food and Drug Safety (MFDS), as an empirical case. The objectives were (1) to describe participant characteristics and teachers’ awareness, use, satisfaction, and perceived barriers regarding government resources in the full survey sample; (2) among teachers with direct MFDS program implementation experience, to estimate topic-specific importance and self-reported implementation gaps and identify relative priorities using the Borich and LF methods; and (3) to examine whether the overall and topic-level gaps differed across school and teacher characteristics. Conceptually, the study links resource dissemination with educational needs prioritization to assess where government provision and classroom implementation may be misaligned. This framework may be relevant to other educational systems that distribute centrally developed resources through teachers.

## 2. Materials and Methods

### 2.1. Study Design and Participants

This cross-sectional study used an online survey of teachers in Korea to assess educational needs related to government-supported school food and nutrition education. Data were collected from October to November 2024. Eligible participants were teachers employed at elementary, middle, and high schools in Korea. Participants were recruited through online teacher communities and official cooperation letters distributed to schools. The study therefore used voluntary, non-probability sampling; no sampling frame was available from which participation probabilities could be calculated.

Data were collected through the KSDC DB platform (Korean Social Science Data Center; https://www.ksdcdb.kr (accessed on 30 August 2024). A total of 1273 completed questionnaires were included. Descriptive analyses of participant characteristics and awareness, use, satisfaction, and barriers regarding MFDS resources used the full sample. The educational needs assessment was restricted to 575 teachers who reported direct implementation of an MFDS-supported food and nutrition education program so that self-reported current implementation would be grounded in program delivery experience. The resulting priority rankings therefore represent experienced implementers and not the wider teaching workforce.

The survey included separate questions on direct program implementation, prior use of MFDS programs or resources, and use of MFDS materials or websites; these indicators were analyzed separately. Questionnaire content was developed for this study as described below. The draft was pilot tested with approximately 30 teachers to identify wording that was unclear or impractical across school levels. The pilot test assessed clarity and practical comprehensibility. The study protocol was approved by the Institutional Review Board of Yongin University (approval No. 2-1040966-AB-N-01-2407-HR-352-1), and all participants provided electronic informed consent before beginning the survey.

### 2.2. Survey Content and Measures

The questionnaire covered five areas: teacher and school characteristics; direct implementation of MFDS-supported food and nutrition education; perceived importance of 32 education topics; self-reported current implementation of the same topics; and awareness, use, satisfaction, and barriers regarding MFDS resources.

The initial topic pool was developed from core food and nutrition education themes in existing MFDS educational materials [24]. The research team consolidated overlapping content into 32 topics spanning foundational nutrition content and contemporary dietary issues. The resulting topic set represents the reviewed MFDS materials rather than an exhaustive or independently validated taxonomy of school food and nutrition education.

Participants rated each topic on two 5-point Likert-type scales. Perceived importance ranged from 1 (not at all important) to 5 (very important), and self-reported current implementation ranged from 1 (not implemented at all) to 5 (implemented very well). The topics were organized descriptively into six content domains: basic nutrition knowledge; dietary behaviors and eating habits; food selection and labeling; food safety and hygiene; health promotion; and contemporary food-related issues. These groupings supported questionnaire organization and were not treated as validated psychometric subscales. The six descriptive domains were not analyzed as latent constructs or subscales; all needs analyses were conducted at the topic level and for the participant level mean across the 32 topics.

Resource measures covered three forms of MFDS provision: a food safety information website, a YouTube channel, and food and nutrition education programs and materials. Functional English descriptors are used in this article, while respondents saw the official Korean resource names. They reported awareness and prior use of each resource. Users rated satisfaction on a 5-point scale, with higher scores indicating greater satisfaction. Separate single-response items assessed the main reason for using or not using MFDS food and nutrition education materials and websites.

### 2.3. Statistical Analysis

Analyses were conducted using IBM SPSS Statistics, version 26.0 (IBM Corp., Armonk, NY, USA). Categorical variables are presented as frequencies and percentages, and continuous variables as mean and standard deviation (SD). In the 575 direct implementers, the participant level gap for topic j was D_ij = I_ij − C_ij, where I denotes perceived importance and C denotes self-reported current implementation. Positive values indicate that importance exceeded implementation. Paired t tests compared I_ij and C_ij. Cohen’s dz was calculated as mean (D_ij)/SD(D_ij). Benjamini–Hochberg false discovery rate adjusted *q* values were calculated for the 32 paired topic comparisons. The Borich score for topic j was B_j = [(sum_i D_ij)/N] × mean(I_j), equivalent to the mean topic gap multiplied by mean topic importance [14]. Larger positive scores indicate higher relative priority within the 32 topics.

In the LF model, mean importance was plotted on the horizontal axis and mean gap on the vertical axis. The grand means of the 32 topic means were used as cut points, with classifications based on unrounded values [16]. LF quadrants and Borich ranks are sample- and scale-dependent descriptive priorities rather than external thresholds. Likert-type ratings were treated as approximately continuous. Paired tests assume independent respondents and approximately normal difference scores; the large implementer sample makes the tests reasonably robust to moderate non-normality.

School level was coded as elementary versus middle/high school. Welch t tests compared overall and topic-level gaps between these groups, and the 32 topic-level *p* values were adjusted using the Benjamini–Hochberg procedure. Exploratory subgroup summaries were calculated by sex, location, school ownership, age, teaching experience, and educational attainment. Participant level mean gap was modeled using ordinary least squares regression with HC3 heteroskedasticity-robust standard errors. The primary model included school level, ownership, location, sex, teaching experience, and educational attainment. Because age and teaching experience were strongly correlated (Spearman rho = 0.857), age group replaced teaching experience in a sensitivity model. School size and school identifiers were not collected; school size and clustering could not be modeled. All tests were two-sided, and *p* < 0.05 was considered statistically significant.

## 3. Results

### 3.1. Sociodemographic and Professional Characteristics of Participants

A total of 1273 teachers were included, of whom 575 (45.2%) reported direct experience implementing an MFDS-supported food and nutrition education program (Table 1). Most participants were women (94.7%), 56.8% worked in middle or high schools, and 92.7% worked in national or public schools. Teachers in their 30s constituted the largest age group (33.6%). School locations included metropolitan or large cities (41.9%), small or medium-sized cities (28.0%), and town or rural areas (30.2%). Nearly half of the participants had 10 or fewer years of teaching experience (49.2%), and 47.4% were enrolled in graduate education or had completed a graduate degree.

### 3.2. Overall Importance–Implementation Profiles of Food and Nutrition Education Topics

Across the 575 direct implementers, the overall mean importance score was 4.42 (*SD* = 0.45), whereas the overall mean self-reported current implementation score was 4.20 (*SD* = 0.61), producing a mean gap of 0.22 (*SD* = 0.41; *t*(574) = 13.05, *p* < 0.001; Table 2). Importance exceeded current implementation for 31 of 32 topics after false discovery rate adjustment; meal planning and menu development was the only nonsignificant topic. Topic-level Cohen’s dz values ranged from 0.061 to 0.556, and dz for the overall mean gap was 0.544. The largest effects were observed for late night eating (dz = 0.556), choosing healthier convenience store foods (dz = 0.475), adolescents and caffeine (dz = 0.475), the importance of nutrition during adolescence (dz = 0.445), and excessive weight control practices (dz = 0.425).

### 3.3. Priority Topics Identified by Borich Needs Assessment

Using the Borich formulation, the five highest scores were observed for problems associated with late night eating (2.01; rank 1), adolescents and caffeine (1.79; rank 2), choosing healthier convenience store foods (1.77; rank 3), excessive weight control practices (1.66; rank 4), and eating disorders (1.57; rank 5; Table 3). Ranks 6 through 10 were lifestyle-related diseases and dietary management (1.48), the importance of nutrition during adolescence (1.48), fruit and vegetable intake (1.33), preferences for sweet and salty tastes (1.13), and food and media information (1.12). The lowest scores were observed for meal planning and menu development (0.20; rank 32), the Dietary Reference Intakes for Koreans and meal planning guidance (0.50; rank 31), and food groups and the Korean Food Balance Wheels (0.61; rank 30). These rankings are relative to the assessed topic set and do not indicate that lower-ranked topics are unimportant.

### 3.4. Prioritization Quadrants in the Locus for Focus Model

**Figure 1:**
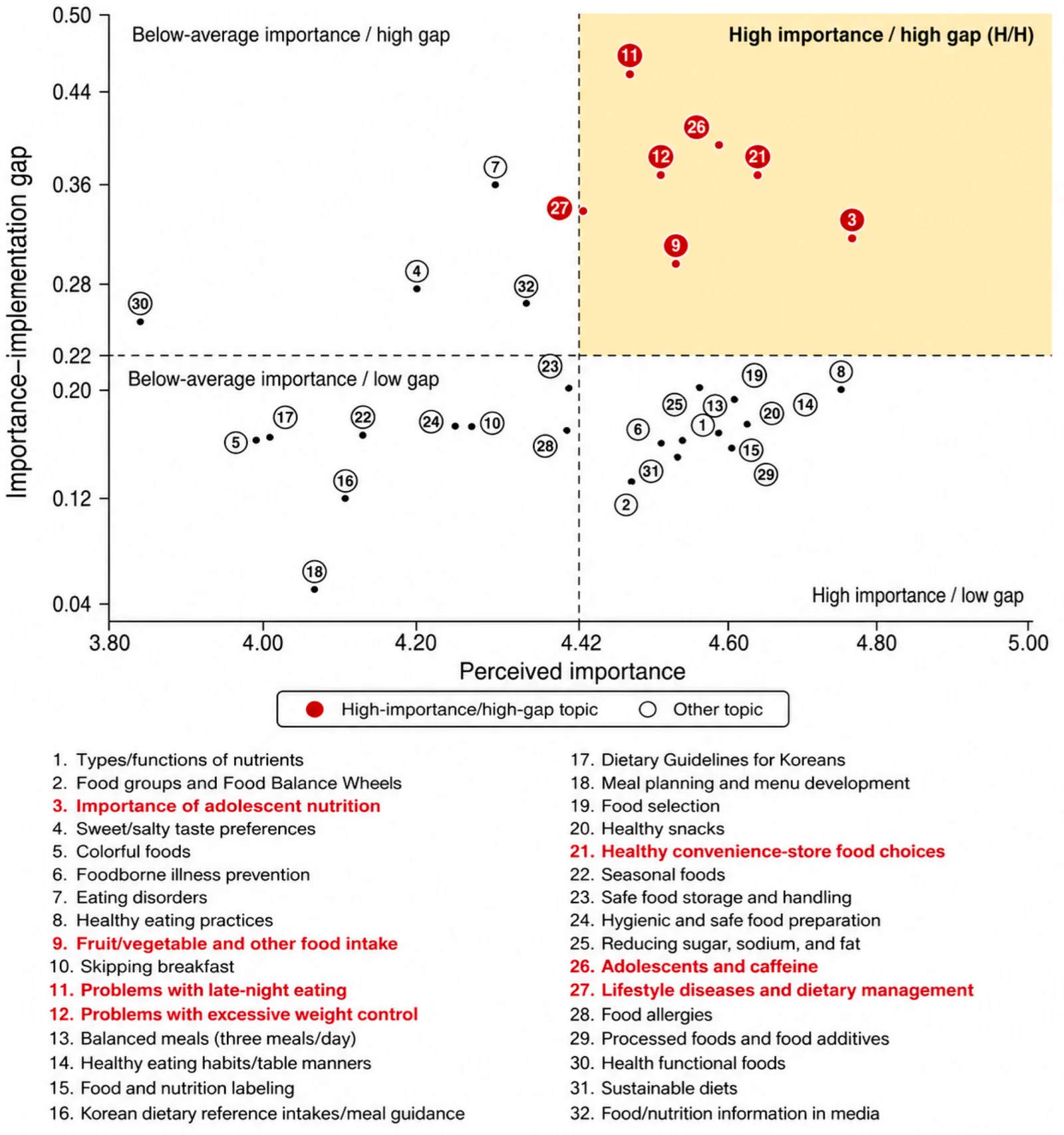
Locus for Focus matrix for prioritizing food and nutrition education topics (*n* = 575). Numbered topic markers correspond to topic labels showed in the two-column key. Dashed lines indicate the unrounded grand means for perceived importance (4.418) and the importance–implementation gap (0.222). Red markers and key entries indicate topics in the relative high-importance/high-gap quadrant. Quadrant assignments were based on unrounded values and represent relative priorities within this sample.

Using the grand mean for importance (4.42) and the grand mean importance–implementation gap (0.22) as cut points, seven topics were located in the high-importance/high-gap quadrant: problems associated with late night eating, adolescents and caffeine, choosing healthier convenience store foods, excessive weight control practices, lifestyle-related diseases and dietary management, the importance of nutrition during adolescence, and fruit and vegetable intake (Figure 1). Quadrant assignment used unrounded values. Four additional topics—preferences for sweet and salty tastes, eating disorders, health functional foods, and food and media information—were located in the below-average-importance/high-gap quadrant. The LF quadrants provide a relative, data-driven screening of the 32 topics; they do not establish an external threshold of practical importance, implementation difficulty, or expected intervention benefit.

### 3.5. School-Level Differences in Importance–Implementation Gaps

School level was analyzed as elementary (*n* = 309) versus middle/high school (*n* = 266). The overall mean gap was 0.284 (*SD* = 0.441) among elementary teachers and 0.149 (*SD* = 0.352) among middle/high school teachers (mean difference = 0.135, 95% CI 0.070 to 0.200; Welch t(569.8) = 4.08, *p* < 0.001; Table 4). After Benjamini–Hochberg adjustment, 20 of the 32 topic comparisons remained significant at *q* < 0.05. Large differences included adolescents and caffeine (0.592 vs. 0.154), the importance of nutrition during adolescence (0.476 vs. 0.120), excessive weight control practices (0.547 vs. 0.158), and breakfast skipping (0.324 vs. 0.000). Meal planning and menu development showed a negative gap among middle/high school teachers (−0.094), indicating that mean implementation slightly exceeded mean importance. These comparisons were exploratory despite multiplicity adjustment.

### 3.6. Awareness, Use, and Satisfaction Regarding Government-Provided Educational Resources

In the full sample (*n* = 1273), awareness and prior use varied across resource types (Table 5). For the MFDS food safety information website, 75.3% were aware of the website and 72.7% had used it; mean satisfaction among users was 3.86 (*SD* = 0.80). For the MFDS YouTube channel, 27.9% were aware of the channel and 24.5% had used it; mean satisfaction among users was 3.85 (*SD* = 0.80). For MFDS food and nutrition education programs, 62.0% reported awareness and 60.7% reported prior use; mean satisfaction was 3.56 (*SD* = 0.83). Separately, 29.8% reported using MFDS food and nutrition education materials or websites. Among these users (*n* = 379), the leading reasons were immediate classroom applicability (29.0%), perceived accuracy and reliability (26.9%), and resource diversity (25.9%). Among nonusers (*n* = 894), the leading reason was lack of awareness that the resources existed (46.4%), followed by difficulty finding immediately applicable materials (17.3%) and a lack of recent updates (11.2%).

### 3.7. Exploratory Subgroup and Multivariable Analyses

Exploratory subgroup and multivariable analyses are presented in Supplementary Tables S1 and S2. In unadjusted analyses, the overall importance–implementation gap differed by school level and teaching experience, whereas no statistically significant differences were observed by sex, school ownership, location, age group, or educational attainment. In the primary HC3 model, middle/high school teachers had a smaller adjusted gap than elementary teachers (B = −0.122, 95% CI −0.197 to −0.047, *p* = 0.001). Some teaching experience categories also had smaller gaps than the 0–5-year group, although the pattern was not monotonic. The association with school level remained in the age group sensitivity model (B = −0.138, 95% CI −0.211 to −0.065, *p* < 0.001). These findings should be interpreted as exploratory.

## 4. Discussion

This study combined a descriptive assessment of government educational resources in the full sample with a topic-level needs assessment among teachers who had directly implemented MFDS-supported programs. Four findings merit emphasis. First, perceived importance exceeded self-reported current implementation for 31 of 32 topics after multiplicity adjustment. Second, the Borich and LF analyses converged on several behaviorally and environmentally relevant priorities. Third, the larger overall gap among elementary teachers remained after multivariable adjustment. Fourth, awareness and use varied substantially by resource type, and lack of awareness and limited classroom applicability were prominent barriers. The overall importance–implementation pattern is consistent with Korean studies reporting gaps in nutrition education tasks [16,17].

Seven topics were located in the high-importance/high-gap quadrant, and the three highest Borich priorities—late night eating, caffeine, and convenience store food selection—were also located in that quadrant. This convergence supports their interpretation as leading priorities identified by experienced teachers. It does not demonstrate that students in the participating schools had corresponding knowledge or behavioral deficits, because student needs and outcomes were not assessed.

The lower-ranked topics are also informative. Meal planning and menu development, the Dietary Reference Intakes for Koreans and meal planning guidance, food groups and the Korean Food Balance Wheels, types and functions of nutrients, and food labeling showed comparatively small gaps. These topics overlap with established food and nutrition content in elementary Practical Arts and, especially, secondary Technology and Home Economics [12]. Lower Borich rankings may therefore reflect more regular curricular opportunities rather than lower educational value. Contemporary risk topics may be less consistently embedded in routine lessons and may require updated, modular resources.

The leading teacher-identified priorities mirror features of the contemporary adolescent food environment. Population studies have associated night eating with breakfast skipping, higher snack energy intake, and lower dietary diversity among Korean adolescents [19,23]. Other studies have documented increases in frequent energy drink consumption [22] and frequent substitution of convenience store foods for meals [21,25]. These findings provide context for why teachers may regard the topics as relevant; they are not direct evidence of the needs of students in the participating schools. Possible educational applications include comparing convenience store meals, interpreting caffeine and nutrition labels, planning alternatives to late night eating, and selecting affordable fruit- and vegetable-containing options.

Eating disorders illustrate why the two prioritization methods should be interpreted together. This topic ranked fifth in the Borich analysis but fell below the LF grand mean for importance, even though its absolute importance rating was high (M = 4.32). Its location outside the high-importance/high-gap quadrant reflects the relative LF cut point rather than a lack of educational relevance. Korean adolescents have reported disordered weight control behaviors, and weight overestimation has been associated with unhealthy dieting and disturbed eating [20,26]. Educational materials should use body image sensitive, non-stigmatizing language and guide teachers to recognize concerns and refer students to appropriate professionals rather than attempt diagnosis or treatment [27,28].

The overall gap was larger among elementary teachers than among middle/high school teachers, and this association persisted after adjustment for location, school ownership, sex, teaching experience, and educational attainment. The difference should not be interpreted automatically as weaker competence or poorer teaching. Several highly discrepant items concerned adolescence, caffeine, eating disorders, or excessive weight control, and elementary schools may provide fewer age relevant curricular opportunities. Food and nutrition education in Korea is also distributed across curriculum-based classes and school-specific arrangements [12,13]. Several teaching experience categories had smaller adjusted gaps than the 0–5-year group, but the pattern was not monotonic and the model explained only a small proportion of variance. These subgroup findings are exploratory. School size and school-level clustering could not be evaluated because those identifiers were not collected.

The resource findings suggest a dissemination and implementation challenge rather than only a shortage of content. Awareness and prior use were relatively high for the MFDS food safety information website but low for the MFDS YouTube channel, and lack of awareness was the most common reason for not using education-specific materials or websites. The 72.7% reporting any prior use of the general food safety website and the 29.8% reporting use of education materials or websites refer to different survey items and should not be treated as interchangeable. Similar resource awareness and curricular barriers have been reported among teachers in California and the Midwestern United States [6,8]. Active promotion, searchable resources, and materials that teachers can adapt directly may therefore be more effective than passive posting. Digital resources could also be integrated into preservice education and continuing professional development through short practice-oriented modules, model lessons, peer exchange, and guided adaptation for different school levels and subject contexts.

The resource findings resemble challenges reported in other educational systems. Australian, Chinese, South African, Cypriot, and Greek studies have described limited instructional time, incomplete preparation, insufficient current materials, limited information, or need for expert support [5,9,10,11,29]. These parallels suggest that visibility, practical usability, and teacher preparation are not unique to Korea. However, the centralized MFDS portfolio, its relationship to the Korean curriculum, and the specific ranking of late-night eating, caffeine, convenience store foods, and weight control topics remain context-dependent.

International implementation research also indicates that educational content is most useful when accompanied by organizational support. In urban Indonesia, competing priorities, nonspecific policy guidance, and support that did not match perceived needs limited readiness for school wide adoption [30]. In Finland, the teacher delivered Tasty School model was feasible, but time remained a barrier and support from principals and colleagues was an important facilitator [31]. A systematic review found variable effects of strategies intended to improve implementation of school healthy eating and physical activity policies and programs [32]. Together, these findings support active dissemination, adaptable lessons, practice-oriented training, and continuing school-level support rather than passive resource provision alone.

Implementation should be measured directly when student benefit is at issue. In the Dutch Krachtvoer program, greater completeness of teacher implementation was associated with improvements in some, but not all, dietary outcomes [33]. In the US Shaping Healthy Choices Program, teacher experience, self-efficacy, and nutrition knowledge were related to implementation fidelity and completeness [34]. These studies reinforce the limitation of the present self-reported implementation measure. The Ethiopian study by Kifle et al. provides relevant evidence about teachers’ perceptions of students’ dietary practices but, like the present teacher survey, cannot substitute for direct assessment of students’ needs or outcomes [35].

Several limitations warrant consideration. Voluntary non-probability sampling limits generalizability. Teachers with greater interest in nutrition education may have been more likely to participate, which could have influenced both importance ratings and reported implementation. Women and teachers in national or public schools were also overrepresented. The needs assessment was restricted to teachers with direct MFDS program experience; teachers without such experience may perceive different priorities or face more fundamental barriers to initiating instruction. Current implementation was self-reported rather than observed and cannot establish the frequency, duration, fidelity, or quality of classroom delivery. Recall and social desirability bias are possible. The study did not assess students, their dietary behavior, or correspondence between teachers’ priorities and objectively measured student needs.

The 32 paired comparisons and 32 school-level topic comparisons increase the opportunity for a type I error. A false discovery rate adjustment was applied, but the subgroup analyses were not prespecified confirmatory tests and remain exploratory. The topic pool was derived from MFDS materials and pilot tested but did not undergo formal expert panel content validation, content validity indexing, or construct validation. The six descriptive domains were not psychometrically validated. Finally, LF classifications depend on sample-specific grand means, and lower relative priority does not imply low absolute importance or low implementation difficulty.

The study nevertheless has several strengths, including a large sample spanning metropolitan, urban, and rural settings, a needs analysis grounded in direct program delivery experience, and complementary use of Borich rankings and the LF matrix. The findings provide a practical basis for revising one government-supported resource portfolio and may inform other systems that disseminate centrally developed materials through teachers. Future studies should use more representative sampling, compare implementers with nonimplementers, formally validate the topic instrument, observe classroom delivery, account for clustering within schools, and examine student knowledge, dietary behavior, and other relevant outcomes.

## 5. Conclusions

Among teachers with direct MFDS program implementation experience, perceived importance exceeded self-reported current implementation for 31 of 32 topics after false discovery rate adjustment. The Borich and LF methods identified late night eating, caffeine, convenience store choices, excessive weight control practices, and other contemporary issues as relative priorities. Elementary teachers reported a larger overall gap than middle/high school teachers, including after multivariable adjustment. Resource use findings indicated that limited visibility and classroom usability remain important barriers. Government agencies should pair developmentally appropriate, classroom-ready materials with active dissemination, practical teacher training, and continuing school-level support. Although the specific priorities and centralized MFDS resource system are context-dependent, the broader need to align resource visibility, curricular fit, teacher preparation, and implementation support may be relevant in other educational systems. These conclusions reflect teachers’ perceptions and should be complemented by objective implementation measures and student outcomes.

## Figures and Tables

**Table 1:** General characteristics of the study participants (*n* = 1273).

Characteristic/Category	*n*	%
Experience implementing MFDS ^1^ food and nutrition education programs		
Yes	575	45.2
No	698	54.8
Sex		
Male	68	5.3
Female	1205	94.7
Age, years		
20–29	191	15.0
30–39	428	33.6
40–49	311	24.4
≥50	343	26.9
School level		
Elementary school	550	43.2
Middle/high school	723	56.8
School ownership/type		
National/public	1180	92.7
General private	80	6.3
Specialized private (e.g., autonomous private and vocational high schools)	13	1.0
School location		
Metropolitan/large city	533	41.9
Small/medium-sized city	356	28.0
Rural area (eup/myeon)	384	30.2
Teaching experience, years		
0–5	349	27.4
6–10	278	21.8
11–15	166	13.0
16–20	167	13.1
21–25	82	6.4
26–30	137	10.8
>30	94	7.4
Highest educational attainment		
Bachelor’s degree	670	52.6
Currently enrolled in or completed graduate coursework	81	6.4
Graduate degree	522	41.0
Total	1273	100.0

^1^ MFDS, Ministry of Food and Drug Safety.

**Table 2:** Perceived importance and current implementation of food and nutrition education topics among teachers with program implementation experience (*n* = 575).

Topic	Importance	Implementation	*t*	*p*	*q*	Cohen’s dz
	*M* (*SD*)	*M* (*SD*)				
Types and functions of nutrients	4.54 (0.63)	4.38 (0.77)	6.69	<0.001	<0.001	0.279
Food groups and the Korean Food Balance Wheels	4.48 (0.71)	4.34 (0.81)	5.02	<0.001	<0.001	0.209
Importance of nutrition during adolescence	4.76 (0.47)	4.45 (0.75)	10.66	<0.001	<0.001	0.445
Awareness of preferences for sweet and salty tastes	4.21 (0.84)	3.94 (0.99)	7.79	<0.001	<0.001	0.325
Colorful foods	3.99 (0.89)	3.83 (1.03)	4.95	<0.001	<0.001	0.206
Foodborne illness prevention (e.g., proper handwashing)	4.52 (0.68)	4.35 (0.82)	5.23	<0.001	<0.001	0.218
Eating disorders	4.32 (0.81)	3.96 (1.04)	9.97	<0.001	<0.001	0.416
Practices for healthy eating	4.74 (0.50)	4.54 (0.69)	7.10	<0.001	<0.001	0.296
Current intake of foods, including fruits and vegetables	4.54 (0.66)	4.24 (0.87)	9.12	<0.001	<0.001	0.380
Skipping breakfast	4.28 (0.83)	4.11 (0.96)	5.31	<0.001	<0.001	0.221
Problems associated with late night eating	4.47 (0.69)	4.03 (0.96)	13.33	<0.001	<0.001	0.556
Problems associated with excessive weight control practices	4.52 (0.70)	4.15 (0.96)	10.20	<0.001	<0.001	0.425
Balanced meals (importance of eating three meals a day)	4.58 (0.63)	4.41 (0.76)	5.70	<0.001	<0.001	0.238
Healthy eating habits (e.g., table manners)	4.66 (0.57)	4.48 (0.70)	6.84	<0.001	<0.001	0.285
Food labeling and nutrition labeling	4.60 (0.58)	4.44 (0.73)	6.01	<0.001	<0.001	0.251
Dietary Reference Intakes for Koreans and meal planning guidance	4.11 (0.86)	3.99 (0.98)	3.78	<0.001	<0.001	0.158
Dietary Guidelines for Koreans	4.01 (0.87)	3.85 (0.99)	5.21	<0.001	<0.001	0.217
Meal planning and menu development	4.07 (0.88)	4.03 (0.94)	1.47	0.141	0.141	0.061
Food selection	4.60 (0.58)	4.41 (0.73)	6.66	<0.001	<0.001	0.278
Healthy snacks	4.62 (0.59)	4.44 (0.75)	6.37	<0.001	<0.001	0.266
How to choose healthier foods at convenience stores	4.61 (0.63)	4.22 (0.92)	11.39	<0.001	<0.001	0.475
Seasonal foods	4.13 (0.80)	3.97 (0.95)	5.16	<0.001	<0.001	0.215
Hygienic and safe food storage and handling	4.41 (0.75)	4.21 (0.89)	6.92	<0.001	<0.001	0.288
Hygienic and safe food preparation	4.26 (0.83)	4.09 (0.95)	5.53	<0.001	<0.001	0.231
Strategies to reduce sugar, sodium, and fat intake	4.57 (0.61)	4.36 (0.79)	7.00	<0.001	<0.001	0.292
Adolescents and caffeine	4.58 (0.63)	4.19 (0.93)	11.38	<0.001	<0.001	0.475
Lifestyle-related diseases and dietary management	4.42 (0.76)	4.09 (0.98)	10.05	<0.001	<0.001	0.419
Food allergies	4.40 (0.73)	4.23 (0.89)	5.83	<0.001	<0.001	0.243
Processed foods (e.g., food additives and high calorie, nutrient poor foods)	4.62 (0.57)	4.47 (0.75)	5.46	<0.001	<0.001	0.228
Health functional foods	3.85 (0.97)	3.60 (1.10)	6.83	<0.001	<0.001	0.285
Sustainable diets	4.54 (0.69)	4.38 (0.83)	5.47	<0.001	<0.001	0.228
Food and media (e.g., food and nutrition information)	4.36 (0.79)	4.10 (0.95)	8.00	<0.001	<0.001	0.334
Overall	4.42 (0.45)	4.20 (0.61)	13.05	<0.001	—	0.544

*M*, mean; *SD*, standard deviation; dz, paired-samples Cohen’s dz; *q*, Benjamini–Hochberg false discovery rate adjusted *p* value.

**Table 3:** Borich needs scores, rankings, and Locus for Focus priority classification among teachers with program implementation experience (*n* = 575).

Topic	Importance–Implementation Gap ^1^	Borich Score ^2^	Rank	LF H/H ^3^
Types and functions of nutrients	0.17	0.75	20	No
Food groups and the Korean Food Balance Wheels	0.14	0.61	30	No
Importance of nutrition during adolescence	0.31	1.48	7	Yes
Awareness of preferences for sweet and salty tastes	0.27	1.13	9	No
Colorful foods	0.16	0.65	29	No
Foodborne illness prevention (e.g., proper handwashing)	0.16	0.74	23	No
Eating disorders	0.36	1.57	5	No
Practices for healthy eating	0.20	0.96	11	No
Current intake of foods, including fruits and vegetables	0.29	1.33	8	Yes
Skipping breakfast	0.17	0.74	21	No
Problems associated with late night eating	0.45	2.01	1	Yes
Problems associated with excessive weight control practices	0.37	1.66	4	Yes
Balanced meals (importance of eating three meals a day)	0.17	0.78	18	No
Healthy eating habits (e.g., table manners)	0.18	0.83	16	No
Food labeling and nutrition labeling	0.16	0.73	24	No
Dietary Reference Intakes for Koreans and meal planning guidance	0.12	0.50	31	No
Dietary Guidelines for Koreans	0.17	0.66	28	No
Meal planning and menu development	0.05	0.20	32	No
Food selection	0.19	0.89	14	No
Healthy snacks	0.18	0.82	17	No
How to choose healthier foods at convenience stores	0.38	1.77	3	Yes
Seasonal foods	0.17	0.69	27	No
Hygienic and safe food storage and handling	0.20	0.88	15	No
Hygienic and safe food preparation	0.17	0.74	22	No
Strategies to reduce sugar, sodium, and fat intake	0.21	0.94	12	No
Adolescents and caffeine	0.39	1.79	2	Yes
Lifestyle-related diseases and dietary management	0.34	1.48	6	Yes
Food allergies	0.17	0.76	19	No
Processed foods (e.g., food additives and high calorie, nutrient poor foods)	0.15	0.70	25	No
Health functional foods	0.24	0.94	13	No
Sustainable diets	0.15	0.69	26	No
Food and media (e.g., food and nutrition information)	0.26	1.12	10	No

^1^ The importance–implementation gap was calculated using unrounded values. ^2^ The Borich score was calculated using the original formulation: mean gap × mean perceived importance. Higher scores indicate higher relative priority within these 32 topics. ^3^ LF H/H indicates the relative high-importance/high-gap quadrant.

**Table 4:** Differences in importance–implementation gaps between elementary and middle/high school teachers (*n* = 575).

Topic	Elementary	Middle/High	Welch t(*df*)	*p*	*q*
	*M* (*SD*)	*M* (*SD*)			
Types and functions of nutrients	0.249 (0.669) ^1^	0.068 (0.471)	3.80 (552.1)	<0.001	<0.001
Food groups and the Korean Food Balance Wheels	0.210 (0.696)	0.049 (0.577)	3.04 (572.2)	0.002	0.007
Importance of nutrition during adolescence	0.476 (0.820)	0.120 (0.460)	6.52 (497.3)	<0.001	<0.001
Awareness of preferences for sweet and salty tastes	0.304 (0.759)	0.229 (0.905)	1.07 (519.3)	0.287	0.383
Colorful foods	0.074 (0.796)	0.267 (0.777)	−2.93 (564.1)	0.004	0.009
Foodborne illness prevention (e.g., proper handwashing)	0.149 (0.723)	0.180 (0.780)	−0.50 (545.3)	0.617	0.681
Eating disorders	0.528 (0.975)	0.173 (0.695)	5.07 (554.4)	<0.001	<0.001
Practices for healthy eating	0.220 (0.754)	0.184 (0.602)	0.63 (569.8)	0.526	0.602
Current intake of foods, including fruits and vegetables	0.298 (0.735)	0.286 (0.806)	0.19 (541.5)	0.853	0.880
Skipping breakfast	0.324 (0.805)	0.000 (0.727)	5.06 (571.7)	<0.001	<0.001
Problems associated with late night eating	0.547 (0.891)	0.335 (0.682)	3.23 (565.4)	0.001	0.004
Problems associated with excessive weight control practices	0.547 (0.975)	0.158 (0.654)	5.69 (542.1)	<0.001	<0.001
Balanced meals (importance of eating three meals a day)	0.265 (0.769)	0.060 (0.636)	3.50 (572.1)	<0.001	0.002
Healthy eating habits (e.g., table manners)	0.146 (0.610)	0.218 (0.648)	−1.37 (548.5)	0.170	0.248
Food labeling and nutrition labeling	0.210 (0.692)	0.098 (0.548)	2.18 (569.1)	0.030	0.048
Dietary Reference Intakes for Koreans and meal planning guidance	0.223 (0.793)	0.004 (0.729)	3.46 (570.5)	<0.001	0.002
Dietary Guidelines for Koreans	0.249 (0.785)	0.068 (0.718)	2.89 (570.8)	0.004	0.010
Meal planning and menu development	0.172 (0.822)	−0.094 (0.734)	4.09 (572.2)	<0.001	<0.001
Food selection	0.265 (0.730)	0.109 (0.644)	2.73 (572.7)	0.007	0.014
Healthy snacks	0.197 (0.616)	0.154 (0.723)	0.77 (523.7)	0.444	0.531
How to choose healthier foods at convenience stores	0.469 (0.839)	0.286 (0.763)	2.75 (571.3)	0.006	0.014
Seasonal foods	0.191 (0.833)	0.139 (0.705)	0.81 (572.9)	0.419	0.531
Hygienic and safe food storage and handling	0.265 (0.761)	0.124 (0.598)	2.49 (568.5)	0.013	0.025
Hygienic and safe food preparation	0.239 (0.850)	0.098 (0.619)	2.31 (558.2)	0.021	0.036
Strategies to reduce sugar, sodium, and fat intake	0.204 (0.694)	0.207 (0.715)	−0.05 (554.8)	0.961	0.961
Adolescents and caffeine	0.592 (0.941)	0.154 (0.572)	6.85 (518.2)	<0.001	<0.001
Lifestyle-related diseases and dietary management	0.414 (0.885)	0.244 (0.682)	2.60 (566.3)	0.010	0.019
Food allergies	0.139 (0.696)	0.211 (0.722)	−1.20 (553.5)	0.230	0.320
Processed foods (e.g., food additives and high calorie, nutrient poor foods)	0.210 (0.701)	0.083 (0.615)	2.33 (572.8)	0.020	0.036
Health functional foods	0.269 (0.858)	0.214 (0.853)	0.76 (561.3)	0.448	0.531
Sustainable diets	0.146 (0.731)	0.162 (0.596)	−0.29 (571.4)	0.772	0.824
Food and media (e.g., food and nutrition information)	0.311 (0.814)	0.195 (0.716)	1.81 (572.7)	0.072	0.109
Overall	0.284 (0.441)	0.149 (0.352)	4.08 (569.8)	<0.001	—

^1^ Values are importance–implementation gaps. School level was grouped as elementary (*n* = 309) versus middle/high school (*n* = 266).

**Table 5:** Teachers’ awareness, use, satisfaction, and perceived barriers regarding MFDS food and nutrition education resources (*n* = 1273).

Item/Response	*n* (%) or *M* (*SD*)
Awareness of the MFDS ^1^ food safety information website	
Yes	958 (75.3)
No	315 (24.7)
Prior use of the MFDS food safety information website	
Yes	926 (72.7)
No	347 (27.3)
Satisfaction with the MFDS food safety information website among users (*n* = 926)	
Satisfaction, *M* (*SD*)	3.86 (0.80) ^2^
Awareness of the MFDS YouTube channel	
Yes	355 (27.9)
No	918 (72.1)
Prior use of the MFDS YouTube channel	
Yes	312 (24.5)
No	961 (75.5)
Satisfaction with the MFDS YouTube channel among users (*n* = 312)	
Satisfaction, *M* (*SD*)	3.85 (0.80)
Awareness of MFDS food and nutrition education programs	
Yes	789 (62.0)
No	484 (38.0)
Prior use of MFDS food and nutrition education programs	
Yes	773 (60.7)
No	500 (39.3)
Satisfaction with MFDS food and nutrition education programs among users (*n* = 773)	
Satisfaction, *M* (*SD*)	3.56 (0.83)
Use of MFDS food and nutrition education materials or websites	
Yes	379 (29.8)
No	894 (70.2)
Reason for using MFDS food and nutrition education materials or websites (*n* = 379)	
To obtain materials and information on desired topics	51 (13.5)
To obtain a wide variety of materials and information	98 (25.9)
To obtain materials and information that could be directly applied in teaching	110 (29.0)
Because the materials and information were accurate and reliable	102 (26.9)
Because current materials and information were updated frequently	5 (1.3)
Because materials and information on the website were easy to access	12 (3.2)
Other	1 (0.3)
Reason for not using MFDS food and nutrition education materials or websites (*n* = 894)	
Was unaware that the educational materials and websites existed	415 (46.4)
Could not find educational materials and information on desired topics	79 (8.8)
Could not find a wide variety of educational materials and information	79 (8.8)
It was difficult to find educational materials and information that could be directly applied in teaching	155 (17.3)
The educational materials and information were perceived as inaccurate or unreliable	3 (0.3)
Current educational materials and information were not updated	100 (11.2)
Materials and information on the website were difficult to access	23 (2.6)
Other (used other resources)	13 (1.5)
Other (heavy workload, lack of time, or did not provide nutrition education)	19 (2.1)
Other	8 (0.9)

^1^ MFDS, Ministry of Food and Drug Safety. Official Korean resource names were displayed in the questionnaire; functional English labels are used here for clarity. ^2^ Satisfaction values are presented as *M* (*SD*); all other values are *n* (%).

## Data Availability

The data presented in this study are available from the corresponding author upon reasonable request. The data are not publicly available because they contain participant level survey information subject to ethical and privacy restrictions.

## Supplementary Materials

The following supporting information can be downloaded at https://www.mdpi.com/article/10.3390/nu18172843/s1, Table S1: Unadjusted subgroup comparisons of participant-level mean perceived importance, self-reported current implementation, and importance–implementation gap scores across the 32 topics among teachers with direct experience implementing MFDS-supported programs (*n* = 575); Table S2: Exploratory multivariable linear regression models of the participant level mean importance–implementation gap across the 32 topics among teachers with direct experience implementing MFDS-supported programs (*n* = 575).

## Abbreviations

The following abbreviations are used in this manuscript:

BH	Benjamini–Hochberg
CI	confidence interval
FDR	false discovery rate
HC3	heteroskedasticity-consistent type 3 standard error
KSDC	Korean Social Science Data Center
LF	Locus for Focus
MFDS	Ministry of Food and Drug Safety
