# Supplementary material for: Teacher-Identified Priorities and Resource Use in Government-Supported School Food and Nutrition Education in Korea
Source: Nutrients. 2026 Aug 30;18(17):2843. doi: 10.3390/nu18172843 (PMC13567653; doi:10.3390/nu18172843)
Supplement: Supplementary file 1 [file nutrients-18-02843-s001.zip › nutrients-4485351-supplementary.pdf]

Supplementary Materials

Supplementary Table S1. Unadjusted subgroup comparisons of participant level mean perceived importance, self-reported current implementation, and importance-implementation gap scores across the 32 topics among teachers with direct experience implementing MFDS-supported programs (*n* = 575).

Characteristic/category	*n*	Perceived importance, *M (SD)*	*p*	Self-reported implementation, *M (SD)*	*p*	Gap, *M (SD)*	*p*
**Sex**							
Male	20	4.217 (0.505)	0.084	4.062 (0.866)	0.487	0.155 (0.592)	0.607
Female	555	4.425 (0.442)		4.201 (0.595)		0.224 (0.400)	
**School level**							
Elementary	309	4.325 (0.456)	<0.001	4.040 (0.598)	<0.001	0.284 (0.441)	<0.001
Middle/high	266	4.527 (0.409)		4.377 (0.563)		0.149 (0.352)	
**School ownership**							
National/public	538	4.409 (0.446)	0.216	4.178 (0.606)	0.089	0.230 (0.412)	0.136
General private	34	4.574 (0.430)		4.471 (0.549)		0.103 (0.328)	
Specialized private	3	4.365 (0.378)		4.312 (0.579)		0.052 (0.201)	
**School location**							
Metropolitan/large city	230	4.433 (0.434)	0.782	4.225 (0.604)	0.572	0.209 (0.376)	0.711
Small/medium city	169	4.413 (0.446)		4.195 (0.589)		0.218 (0.399)	
Rural	176	4.403 (0.463)		4.160 (0.625)		0.243 (0.455)	
**Age group**							
20-29 years	93	4.457 (0.381)	0.397	4.155 (0.553)	0.335	0.302 (0.389)	0.084
30-39 years	190	4.435 (0.443)		4.214 (0.624)		0.221 (0.440)	
40-49 years	133	4.430 (0.421)		4.262 (0.589)		0.168 (0.373)	
50 years or older	159	4.366 (0.500)		4.144 (0.626)		0.221 (0.402)	
**Teaching experience**							
0-5 years	163	4.472 (0.399)	0.029	4.156 (0.556)	0.012	0.316 (0.440)	0.002
6-10 years	133	4.405 (0.449)		4.212 (0.636)		0.192 (0.406)	
11-15 years	66	4.459 (0.424)		4.362 (0.559)		0.097 (0.300)	
16-20 years	70	4.478 (0.438)		4.288 (0.634)		0.190 (0.401)	
21-25 years	30	4.344 (0.506)		4.166 (0.536)		0.178 (0.315)	
26-30 years	71	4.243 (0.484)		3.974 (0.666)		0.269 (0.421)	
>30 years	42	4.435 (0.487)		4.284 (0.567)		0.151 (0.414)	
**Educational attainment**							
Bachelor's degree	293	4.393 (0.448)	0.026	4.165 (0.621)	0.047	0.227 (0.398)	0.481

**Characteristic/category**	***n***	**Perceived importance, *M (SD)***	***p***	**Self-reported implementation, *M (SD)***	***p***	**Gap, *M (SD)***	***p***
Graduate enrollment/coursework	29	4.592 (0.362)		4.443 (0.559)		0.149 (0.329)	
Graduate degree	253	4.428 (0.449)		4.204 (0.589)		0.224 (0.428)	

Values are mean (*SD*) unless otherwise indicated. The gap was calculated as perceived importance minus self-reported current implementation; positive values indicate that perceived importance exceeded implementation. The *p* values are unadjusted, exploratory omnibus tests and are shown once per characteristic. Two-category variables were compared using Welch t tests, and variables with more than two categories were compared using Welch ANOVA. School size was not collected and could not be examined. *M*, mean; *SD*, standard deviation.

Supplementary Table S2. Exploratory multivariable linear regression models of the participant level mean importance-implementation gap across the 32 topics among teachers with direct experience implementing MFDS-supported programs (*n* = 575).

Predictor	B	HC3 SE	95% CI	*p*
**Primary model including teaching experience**				
Intercept	0.380	0.045	0.292 to 0.469	<0.001
Middle/high school	-0.122	0.038	-0.197 to -0.047	0.001
Private school	-0.035	0.063	-0.158 to 0.088	0.578
Small/medium city	-0.004	0.040	-0.082 to 0.074	0.918
Rural area	0.000	0.042	-0.082 to 0.082	0.996
Male	-0.050	0.142	-0.328 to 0.229	0.726
Teaching experience: 6-10 years	-0.125	0.051	-0.224 to -0.025	0.014
Teaching experience: 11-15 years	-0.194	0.055	-0.303 to -0.086	<0.001
Teaching experience: 16-20 years	-0.121	0.065	-0.249 to 0.006	0.062
Teaching experience: 21-25 years	-0.144	0.067	-0.275 to -0.014	0.031
Teaching experience: 26-30 years	-0.088	0.062	-0.210 to 0.034	0.159
Teaching experience: >30 years	-0.184	0.074	-0.329 to -0.039	0.013
Graduate enrollment/coursework	-0.017	0.072	-0.159 to 0.125	0.810
Graduate degree	0.004	0.039	-0.072 to 0.080	0.916
**Sensitivity model including age group**				
Intercept	0.385	0.051	0.285 to 0.485	<0.001
Middle/high school	-0.138	0.037	-0.211 to -0.065	<0.001
Private school	-0.032	0.059	-0.147 to 0.083	0.583
Small/medium city	0.003	0.039	-0.074 to 0.081	0.929
Rural area	0.006	0.043	-0.078 to 0.089	0.895
Male	-0.045	0.141	-0.322 to 0.232	0.749
Age: 30-39 years	-0.097	0.054	-0.203 to 0.009	0.074
Age: 40-49 years	-0.153	0.063	-0.276 to -0.029	0.016
Age: 50 years or older	-0.132	0.054	-0.238 to -0.025	0.015
Graduate enrollment/coursework	-0.031	0.071	-0.169 to 0.108	0.666
Graduate degree	0.016	0.043	-0.067 to 0.100	0.701

The outcome was the participant-level mean importance-implementation gap across the 32 topics, calculated as perceived importance minus self-reported current implementation. B values are un-standardized coefficients and represent adjusted mean differences relative to the reference category. Both models used HC3 heteroskedasticity-robust standard errors. Primary model included school level, school ownership, location, sex, teaching experience, and educational attainment. Sensitivity model replaced teaching experience with age group. Reference categories were elementary school, national/public school, metropolitan/large city, female, 0-5 years of teaching experience, and bachelor's degree; the age reference in sensitivity model was 20-29 years. Model fit was R2 = 0.057 (adjusted R2 = 0.035) for primary model and R2 = 0.043 (adjusted R2 = 0.026) for sensitivity model. All analyses were exploratory. B, unstandardized coefficient; CI, confidence interval; HC3, heteroskedasticity-consistent type 3 standard error; R2, coefficient of determination.
